# Safety and efficacy of scissor-type knives in colorectal endoscopic submucosal dissection: International multicenter observational study

**DOI:** 10.1055/a-2733-0944

**Published:** 2025-11-11

**Authors:** Kuilang Liu, Jing Wu, Yuzuru Tamaru, Yadan Wang, Hui Su, Chunmei Guo, Canghai Wang, Hong Liu, Makoto Kobayashi, Kiyoaki Honma, Takuya Yamada, Levchenko Evgeniy, Noor Mohammed, Sergio Cadoni, Adolfo Parra-Blanco, Antipova Mariya, Sauid Ishaq, Toshio Kuwai

**Affiliations:** 1 117968 Gastroenterology, Beijing Shijitan Hospital Capital Medical University, Beijing, China; 2 26455 Gastroenterology, Capital Medical University Affiliated Beijing Friendship Hospital, Beijing, China; 3 37086 Department of Gastroenterology, Kure Medical Center and Chugoku Cancer Center, Kure, Japan; 4 37036 Gastroenterology, Yokkaichi Municipal Hospital, Yokkaichi, Japan; 5 157373 Nihonkai General Hospital, Sakata, Japan; 6 13824 Department of Gastroenterology, Osaka Rosai Hospital, Sakai, Japan; 7 Endoscopy Department, City Hospital No. 2, St. Petersburg, Russia; 8 4472 Gastroenterology, Leeds Teaching Hospitals NHS Trust, Leeds, United Kingdom of Great Britain and Northern Ireland; 9 Digestive Endoscopy Unit, CTO Hosptital, Iglesias, Italy; 10 9820 NIHR Nottingham Digestive Diseases Biomedical Research Unit, Nottingham University Hospitals NHS Trust, Nottingham, United Kingdom of Great Britain and Northern Ireland; 11 Saint Petersburg State Budgetary Institution of Healthcare, City Mariinskaya Hospital, St. Petersburg, Russia; 12 1722 Aston University, Aston University, Birmingham, United Kingdom of Great Britain and Northern Ireland; 13 7714 Health and science, Dudley Group of Hospitals NHS Trust, Dudley, United Kingdom of Great Britain and Northern Ireland; 14 68272 Gastrointestinal Endoscopy and Medicine, Hiroshima University Hospital, Hiroshima, Japan

**Keywords:** Endoscopy Lower GI Tract, Colorectal cancer, Polyps / adenomas / ..., Endoscopic resection (polypectomy, ESD, EMRc, ...)

## Abstract

**Background and study aims:**

Scissor-type knives have shown safety and efficacy in endoscopic submucosal dissection (ESD) procedures, particularly in studies from Japan. However, the safety and efficacy of these devices in international settings, particularly outside Japan, is not well established.

**Patients and methods:**

This was a prospective, multicenter, observational study conducted across nine international centers, encompassing a total of 461 lesions from 460 patients. In subgroup analysis, 162 lesions came from four institutions in Japan (Japanese institutions group, JAG) and 299 lesions from five institutions outside Japan (non-Japanese institutions group [NJAG]). After 1:1 propensity score matching resulted in 120 matched pairs of lesions, key outcomes were compared between groups.

**Results:**

The overall perforation rate during ESD procedures was 0.87%. Intraoperative perforations were observed more frequently in NJAG than JAG (3 vs. 1 event, 1.9% vs. 0.33%, respectively), although not statistically significant ( *P* = 0.127). Overall incidence of delayed bleeding was also 0.87%, with no delayed bleeding reported in NJAG. Post propensity matching analysis revealed a significantly slower median resection speed in NJAG compared with JAG (9.12 0.86–56.57 vs 26.21 1.95–93.54 mm²/min, *P* < 0.001). Both histological complete resection and curative resection rates were significantly lower in NJAG than in JAG with rates of 88.3% vs 98.3% for histological complete resection and 83.3% vs 95% for curative resection (both *P* < 0.01).

**Conclusions:**

Use of scissor-type knives in colorectal ESD outside Japan demonstrated a favorable safety profile. However, certain performance outcomes, such as resection speed and resection success rates, were inferior to Japanese institutions.

## Introduction

Endoscopic submucosal dissection (ESD), initially developed in Japan, offers a significant advantage in achieving curative resection irrespective of tumor size or shape. ESD is gaining attraction for the removal of early colorectal neoplasms in both Eastern and Western countries 1 2 .

However, colorectal ESD presents unique challenges compared with ESD for the esophagus and stomach, particularly in non-Japanese settings where expertise and training may be limited 1 3 . Studies have indicated that outcomes of colorectal ESD in non-Asian region often lag behind those reported in Asia, with lower en bloc resection rates and higher incidence of adverse events (AEs) 2 4 . Consequently, the American Gastroenterological Association has recommended that colorectal ESD procedures be performed at high-volume, specialized centers to enhance safety and improve clinical outcomes 5 . Conventional ESD devices, such as the Dual Knife/J (Olympus Co., Tokyo, Japan) and the Flush Knife/BT (Fujifilm, Inc, Tokyo, Japan) 6 7 , feature uninsulated tips that requires scope rotation and precise manipulation. This can complicate colorectal ESD procedures due to colon’s intricate complex folds and thin walls, necessitating advanced endoscopic skills to manage these devices effectively 6 . To mitigate these challenges and reduce the need for advanced maneuvering skills, several scissor-type knives, such as the SB knife (Sumitomo Bakelite Co., Tokyo, Japan) and the clutch cutter knife (Fujifilm, Inc, Tokyo, Japan), have been developed. These devices aim to simplify ESD procedures 8 9 10 11 12 13 . Studies conducted in Japan have demonstrated that scissor-type knives provide safer resections and lower perforation rates than traditional needle-type knives 14 15 . However, the efficacy and safety of these scissor-type knives for resecting early colorectal neoplasm outside Japan remains less well known. To investigate this, an international, multicenter observational study was designed to evaluate the performance, safety and efficacy of these knives in diverse clinical settings.

## Material and methods

### Study design

This international, multicenter, prospective observational study included nine centers, including four Japanese institutions (NHO Kure Medical Center and Chugoku Cancer Center, Yokkaichi Municipal Hospital, Homma Hospital, and Osaka Rosai Hospital); one Chinese institution (Beijing Shijitan Hospital, Capital Medical University); two Russian institutions (City Mariinskaya Hospital, St. Petersburg State Budgetary Healthcare Institution); one Italian institution (CTO Hospital, Iglesias, Italy); and one UK institution (St James's University Hospital, Leeds Teaching Hospitals NHS Trust, Leeds, UK).

The ethics committee for each participating institution approved the study in compliance with Declaration of Helsinki ethical principles. This study was registered in 2018 with the University Hospital Medical Network Clinical Trials Registry under the identifier UMIN000031511, and informed consent was obtained from all participants.

### Inclusion and exclusion criteria

In this study, indications for colorectal ESD followed the Japanese Gastroenterological Endoscopy Society guidelines for colorectal ESD indications 16 . Eligible patients met the following criteria: aged 20 to 90 years with laterally spreading, superficial colorectal tumors exceeding 20 mm in diameter and tumors which are difficult to remove en bloc using endoscopic mucosal resection.

In addition, patients also met criteria of Eastern Cooperative Oncology Group performance status of 0–2. Exclusion criteria included: 1) pregnancy; 2) age under 18 years; and 3) any other conditions deemed unsuitable by investigators, based on their clinical judgment.

### ESD procedure and operator experience

**Fig. 1:**
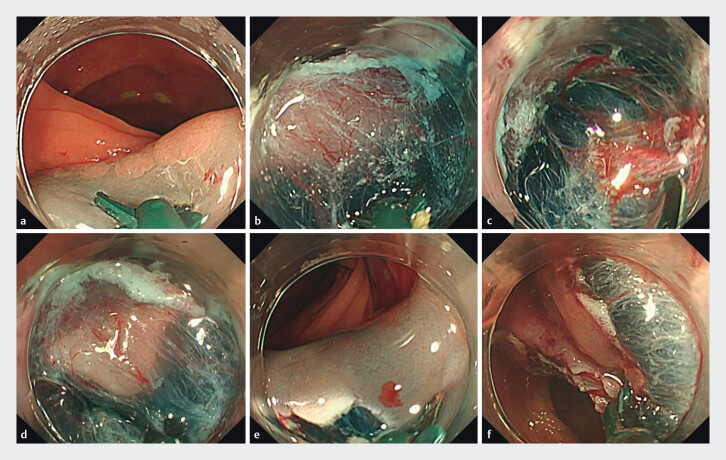
Standard procedure of colorectal endoscopic submucosal dissection using a scissor-type knife (SB Knife Jr). **a** Mucosal incision from the anal side. **b** Submucosal layer is entered using SB Knife. **c** The vessel is being grasped using SB Knife instead of hemostatic forceps. **d** Submucosal dissection is advanced using grasping, pulling and cutting method. **e** The lateral margin of the lesion is resected with a blade of SB Knife in the submucosal space. **f** En-bloc resection is achieved.

In this study the SB Knife Jr. was designated as the primary device across all centers, however switching to other devices when necessary was permitted. Snaring resection without submucosal dissection was not classified as ESD. The ESD procedure was generally performed under conscious sedation or general anesthesia. A standard or therapeutic colonoscope or gastroscope equipped with a transparent hood to facilitate mucosal traction was used, based on the practice of each participating center. CO _2_ insufflation and water-jet fluid solutions were employed during scope insertion and the ESD procedure. Submucosal lifting solutions were also applied determined by each center’s standard practice. The scissor-type knife (Stag-Beetle Knife Jr or Jr2, Sumitomo Bakelite, Tokyo, Japan) was used for both dissection and hemostasis, with hemostatic forceps available if required. The standard procedure for colorectal ESD using a scissor-type knife (SB Knife Jr) is presented in Fig. 1 . Electrosurgical unit settings were selected based on manufacturer recommendations or local guidelines. Resected specimens were pinned and fixed on cork for pathological examination. All cases were performed by a total of 13 operators who had performed > 1000 colonoscopies and > 10 gastrointestinal ESDs. All operators were classified as non-experts, having performed fewer than 50 prior ESD procedure, except 3 operators from Japanese institutions.

### Study outcomes and definitions

The primary outcome was incidence of intraoperative perforation, defined as visualization of intraperitoneal adipose tissue due to injury to the muscle layer during the procedure. Secondary outcomes included procedure time, resection outcomes (en bloc, histological complete, and curative resection rates), and incidence of post-procedure AEs such as delayed bleeding and perforation. Procedure time was measured from submucosal injection to completion of lesion resection. Resection speed was calculated by dividing lesion area (measured after resection) by procedure time. En bloc resection was defined as resection of the lesion in a single block, whereas histological complete resection required both en bloc resection and negative horizontal and vertical margins in histological examination. Curative resection was defined as complete resection with the following histological conditions: 1) papillary or tubular adenocarcinomas; 2) a mucosal lesion or submucosal invasion depth < 1000 µm; 3) no lymphovascular invasion; and 4) a tumor-budding grade of 1 (low grade), following guidelines from the Japanese Society for Cancer of the Colon and Rectum (JSCCR) 17 . Delayed bleeding was defined as a hemoglobin drop of more than 2 g/dL or clinical signs (hematochezia or melena) that necessitated unplanned hospital admission, blood transfusion, or unplanned endoscopic or surgical intervention. Delayed perforation was identified by the sudden onset of symptoms and imaging-confirmed free air in the abdomen following an initial uneventful ESD.

### Sample size

Before the start of this study, the sample size was calculated as below: the anticipated perforation rate (p) was estimated at 1% (0.01) according to a study 18 . This rate was estimated with a 95% confidence interval (CI) and a margin of error (d) of ±1% (0.01). Based on these parameters (p = 0.01, d = 0.01, and a 95% CI), the calculated sample size was: 381 lesions. To account for potential patient dropouts or cases with incomplete data, the target sample size was set at 400.

### Statistical analysis

Data for the study collected from all participating centers using electronic case report forms (eCRF) and regular online study monitoring activities were implemented for ensuring data quality and consistency. Statistical analysis was conducted with SPSS software (version 26.0.0.0, released 2019, IBM Corp, Armonk, New York, United States). Continuous variables were expressed as either mean ± standard deviation or median (range), based on data distribution, and analyzed using either the Student’s *t* test for normally distributed date or the Mann-Whitney U test for non-normally distributed data. Categorical variables (including bivariate and multivariate) were presented as percentages and analyzed using the χ² test or Fisher’s exact test depending on sample size. To identify risk factors for adverse outcomes and resection status, both univariate and multivariate binary logistic regression analyses were conducted. Results of multivariate analysis were expressed as odds ratios (ORs) with 95% CI. *P* < 0.05 indicated statistically significance. To address potential selection biases, propensity score matching (PSM) was employed using SPSS 26.0 software to balance baseline characteristics between groups. The variables included in the matching process were age, sex, lesion location, lesion size, growth type, submucosal fibrosis, and pathology. A 1:1 matching was applied using a caliper width of 0.02 to ensure comparability between the two groups.

## Results

### Baseline characteristics of patients and lesions before and after propensity score matching

A total of 461 lesions in 460 patients were included in the analysis. Median age of patients was 70 years (range 32–91 years), with 57.2% being male. Median tumor size was 30 mm (range 10–100 mm). Most lesions (43.6%) were found in the right-sided colon. The most prevalent lesion type was laterally spreading tumor-granular type (LST-G), which accounted for 38.6% of the cases. On histopathological evaluation, adenomas and sessile serrated lesions represented 51.8% of the lesions, whereas 37.3% of lesions were cancers confined to the mucosa or superficial submucosa. In subgroup analysis, 299 lesions in 298 patients were from four Japanese institutions (Japanese institutions group, JAG) and 162 lesions in 162 patients were from five institutions outside Japan (non-Japanese institutions group, NJAG) ( Table 1 ). JAG: compared to NJAG: 27.5 mm (range 10–73 mm; *P* < 0.01). especially in JAG (JAG: 51.2% vs NJAG: 2 9.6%, *P* < 0.001). Median tumor size was 30 mm (10–100 mm) in JAG and 27.5 mm (range 10–73 mm) in NJAG ( *P* < 0.01). The percentage of lesions found in the right-sided colon was higher in JAG than in NJAG (51.2% vs 29.6%, *P* < 0.001).

**Table:** **Table 1** Number of patients and lesions at the nine participating institutions.

	**1**	**2**	**3**	**4**	**5**	**6**	**7**	**8**	**9**
Country	Japan	Japan	Japan	Japan	Italy	Russia	Russia	UK	China
No. of patients	258	28	9	8	4	16	306	16	97
No. of lesions	259	28	9	8	4	16	306	6	97

**Fig. 2:**
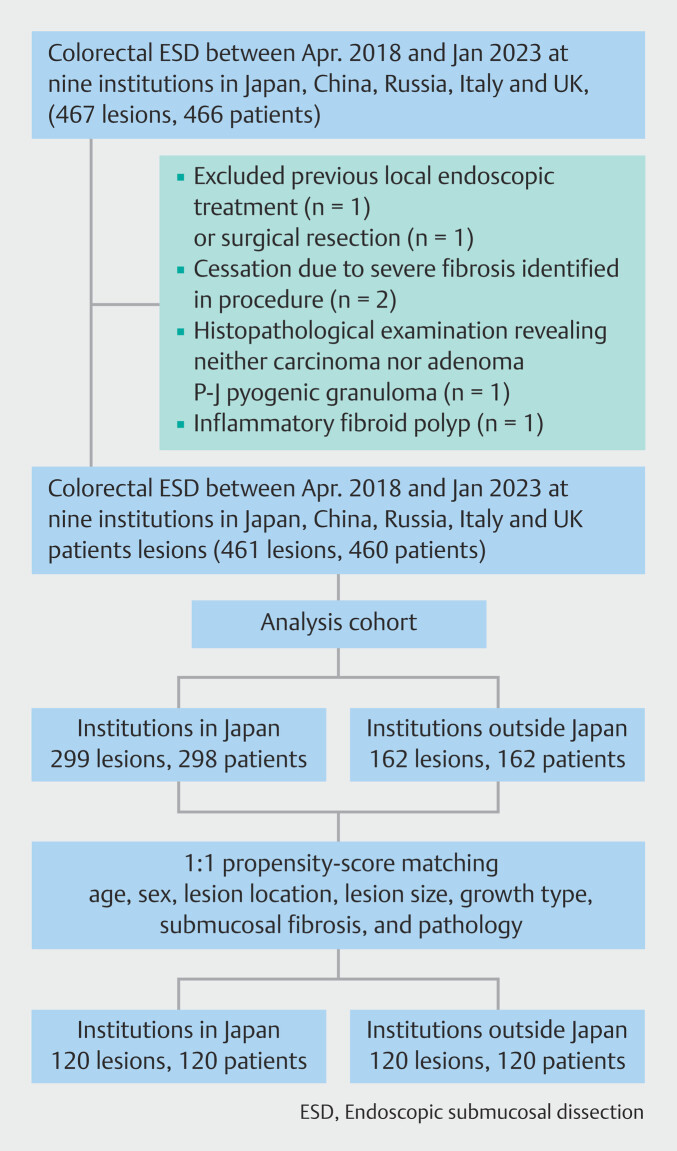
Flowchart of enrolled patients.

In subgroup analysis, to account for differences in baseline characteristics of patients and lesions between the Japanese and Non-Japanese institution groups, 1:1 PSM was conducted. Following this process, 120 lesion pairs were successfully matched from both groups ( Fig. 2 ). After matching, there were no significant differences in baseline characteristics between the two groups. This balance ensured that both groups were comparable, allowing for unbiased analysis in subsequent statistical evaluations. Baseline characteristics of patients and lesions before and after PSM are presented in Table 2 .

**Table:** **Table 2** Characteristics of patients and lesions before and after propensity score–matching.

	**All lesions**					**Propensity score-matched lesions**				
	**Total**	**Institutions in Japan**	**Institutions outside Japan**	***P* value **	**SMD**	**Total**	**Institutions in Japan**	**Institutions outside Japan**	***P* value **	**SMD**
No. of patients	460	298	162			240	120	120		
No. of lesions	461	299	162			240	120	120		
Sex, male	263 (57.2%)	17 4 (58.4%)	89 (54.9%)	0.5	0.073	146	68 (56.7%)	78 (65%)	0.186	0.171
Median age, Y (range)	70 (32–91)	72 (32–90)	65 (37–88)	< 0.001	0.551	67 (32–89)	68 (32–89)	67 (43–88)	0.155	0.011
Median tumor size, mm (range)	30 (10–100)	30 (10–100)	27.5 (10–73)	< 0.001	0.46	28 (10–90)	3 0 (10–90)	28 (10–73)	0.519	0.087
Tumor location				< 0.001	0.462				0.831	0.079
Right-sided colon	201 (43.6%)	153 (51.2%)	48 (29.6%)			76	40 (33.3%)	36 (30%)		
Left-sided colon	117 (25.4%)	70 (23.4%)	47 (29.0%)			64	32 (26.7%)	32 (26.7%)		
Rectum	143 (31.0%)	76 (25.4%)	67 (41.4%)			100	48 (40%)	52 (43.3%)		
Growth type				< 0.001	0.357				0.95	0.041
Laterally spreading tumor-granular	178 (38.6%)	132 (44.1%)	46 (28.4%)			82	41 (34.2%)	41 (34.2%)		
Laterally spreading tumor-nongranular	155 (33.6%)	97 (32.4%)	58 (35.8%)			88	43 (35.8%)	45(37.5%)		
Polypoid	128 (27.8%)	70 (23.4%)	58 (35.8%)			70	36(30%)	34(28.3%)		
Submucosal fibrosis				0.001	0.567				0.473	0.1
None or mild	395 (85.7%)	244 (81.6%)	151 (93.2%)			221	112 (93.3%)	109 (90.8%)		
Severe	66 (14.3%)	55 (18.4%)	11 (6.8%)			19	8 (6.7%)	11 (9.2%)		
Histopathology				0.025	0.479				0.823	0.36
Adenoma (SSL included)	239 (51.8%)	143 (47.8%)	96 (59.3%)			134	66 (55%)	68 (56.7%)		
Tis	146 (31.7%)	96 (32.1%)	50 (30.9%)			78 (32.5%)	41 (34.2%)	37 (30.8%)		
T1a	26 (5.6%)	20 (6.7%)	6 (3.7%)			13 (5.4%)	7 (5.8%)	6 (5%)		
T1b	50 (10.8%)	40 (13.4%)	10 (6.2%)			15 (6.3%)	6 (5%)	9 (7.5%)		
SMD, standard mean difference; SSL, sessile serrated lesion; Tis, intramucosal carcinoma; T1a, submucosal superficial invasive carcinoma less than 1000 μm; T1b, submucosal superficial invasive carcinoma over 1000 μm.										

### Procedure outcome

Procedure outcomes of all ESDs are presented in Table 3 . Median procedure time was 57 minutes (range 10–358), median resection speed was 18.42 mm ^2^ /min (range 0.86–164.04) and rates of en bloc resection, histological complete resection, and the curative resection rate were 97.6%, 93.9%, and 84.4%, respectively. In subgroup analysis, median procedure time was significantly longer in NJAG (82 minutes [range 25–345] vs JAG 50 minutes [range 10–358]; *P* < 0.001). This difference became more pronounced after PSM (83.5 minutes [range 25–345] vs 41.5 minutes [range 10–253]; *P* < 0.001), respectively. Similarly, median resection speed was significantly slower in NJAG (8.83 mm²/min [range 0.86–56.57]) than in JAG 25.17 mm²/min (range 1.95–164.04; *P* < 0.001). This trend persisted after matching (9.12 vs. 26.21 mm²/min, *P* < 0.001). The histological complete resection rate was also significantly lower in NJAG compared with JAG, both before (88.9 vs 96.7%, *P* < 0.01) and after PSM (88.3 vs 98.3%, *P* < 0.01). Although no initial significant difference was seen in the curative resection rate between the groups, post-matching results showed significantly lower rate in NJAG (83.3 vs 95%, *P* < 0.01). Device change did not occur in all procedures except 13 lesions (8.0%) in NJAG switching to hybrid resection using snares.

**Table:** **Table 3** Procedure outcome and adverse events before and after propensity score–matching.

	**All lesions**				**Propensity score-matched lesions**			
	**Total**	**Institutions** **in Japan**	**Institutions** **outside Japan**	***P* value **	**Total**	**Institutions** **in Japan**	**Institutions** **outside Japan**	***P* value **
No. of patients	460	298	162		240	120	120	
No. of lesions	461	299	162		240	120	120	
Procedure time (min)	57 (10–358)	50 (10–358)	82 (25–345)	< 0.001	57.5 (10–345)	41.5 (10–253)	83.5 (25–345)	< 0.001
Resection speed (mm ^2^ /min)	18.42 (0.86–164.04)	25.17 (1.95–164.04)	8.83 (0.86–56.57)	< 0.001	16.48 (0.86–93.54)	26.21 (1.95–93.54)	9.12 (0.86–56.57)	< 0.001
Resection method				< 0.001				< 0.001
En bloc	450 (97.6%)	293 (98.0%)	144 (88.9%)		224 (93.3%)	119 (99.2%)	105 (87.5%)	
Hybrid	13 (27.82%)	0 (0%)	13 (8.0%)		10 (4.2%)	0 (0%)	10 (8.3%)	
Piecemeal	11 (2.4%)	6 (2%)	5 (3.1%)		6 (2.5%)	1 (0.8%)	5 (4.2%)	
Histologic complete resection	433 (93.9%)	289 (96.7%)	144 (88.9%)	0.001	224 (93.3%)	118 (98.3%)	106 (88.3%)	0.002
Curative resection	389 (84.4%)	253 (84.6%)	136 (84.0%)	0.851	214 (89.2%)	114 (95%)	100 (83.3%)	0.004
Adverse events								
Intraoperative perforation	4 (0.87%)	1 (0.33%)	3 (1.9%)	0.094	3 (1.25%)	0 (0%)	3 (2.5%)	0.081
Required surgery	1 (0.22%)	1 (0.33%)	0 (0%)	0.461	0 (0%)	0 (0%)	0 (0%)	
Delayed perforation	1 (0.22%)	0	1 (0.62%)	0.174	1 (0.42%)	0 (0%)	1 (0.83%)	0.5
Required surgery	1 (0.22%)	0	1 (0.62%)	0.174	1 (0.42%)	0 (0%)	1 (0.83%)	0.5
Delayed bleeding	4 (0.87%)	4 (1.34%)	0 (0%)	0.139	2 (0.85%)	2 (1.7%)	0 (0%)	0.249

### Adverse events

The overall rate of intraoperative perforation was 0.87% (4 of 461), with only one case (0.22%, 1 of 461) requiring surgical intervention. In subgroup analysis, incidence of intraoperative perforation was higher in NJAG at 1.9% compared with 0.33% in JAG, but this difference did not reach statistical significance ( *P* = 0.127). All three perforations in NJAG were managed endoscopically, and curative resection was achieved in each case. In addition, there was one case of delayed perforation reported in NJAG. This patient presented with fever and abdominal distension approximately 6 hours following an uneventful ESD of a protruding lesion in the ascending colon, despite clipping of the mucosal defect. An emergency computed tomography (CT) scan confirmed the delayed perforation. Emergency surgery was subsequently performed, which included local resection with lymph node dissection. During surgery, a linear tear in the ulcerated area beneath the clips was identified. Pathological examination of the ESD specimen revealed pT1b adenocarcinoma with a positive vertical margin, although no residual tumor or lymphatic invasion was found in the surgical specimen. The patient recovered well after surgery and was monitored for 5 years without any recurrence. In terms of delayed bleeding, the overall incidence was also recorded at 0.87% (4 of 461 cases) in the entire cohort. Notably, there were no reports of delayed bleeding in the NJAG.

### Risk factors for adverse events and outcomes

In the multivariate analysis conducted on data including all lesions (prior to PSM), no significant risk factors were identified for intraoperative perforation, delayed perforation, or delayed bleeding, likely due to the low frequency of these events. However, several risk factors were associated with incomplete resection, as detailed in Table 4 . The identified risk factors included treatment at a non-Japanese institution, slower resection speed, severe submucosal fibrosis, and a pathology result of T1b. ORs for these factors were as follows: 1) institution outside Japan, OR 8.435 (95% CI 2.487–28.613; *P* < 0.001); 2) slower resection speed, OR 5.204 (95% CI 1.095–24.734; *P* < 0.05); 3) severe submucosal fibrosis, OR 7.657 (95% CI 2.355–24.891; *P* < 0.01); and 4) pathology of T1b, OR 4.508 (95% CI 1.517–13.398; *P* < 0.01).

**Table:** **Table 4** Risk factors for incomplete resection.

Variable	Incomplete resection			
	Univariate analysis		Multivariate analysis	
	OR (95%CI)	*P* value	OR (95%CI)	*P* value
Institutions (non-Japanese institution versus Japanese institution)	3.612 (1.626–8.027)	0.002	8.435 (2.487–28.613)	0.001
Resection speed (≤ 20 mm2/min versus > 20 mm2/min)	11.671 (2.725–49.994)	0.001	5.204 (1.095–24.734)	0.038
Submucosal fibrosis (severe versus mild/none)	6.214 (2.802–13.779)	< 0.001	7.657 (2.355–24.891)	0.001
Pathology (T1b versus adenoma/Tis/T1a)	5.458 (2.360–12.627)	< 0.001	4.508 (1.517–13.398)	0.007

## Discussion

The overall perforation rate in this study was 0.87%, with a corresponding incidence of delayed bleeding that was also 0.87%, indicating no significant difference between NJAG and JAG in subgroup analysis. However, the rate of histological complete resection was significantly lower in NJAG compared with JAG, both before PSM (88.9% vs 96.7%; *P* < 0.01) and after matching (88.3% vs 98.3%; *P* < 0.01). In addition, resection speed in the NJAG group was less than half that in the JAG group.

The study reports a perforation rate of 0.87% with scissor-type knives, which is consistent with prior studies (0%–1%) 11 14 19 , and is notably lower than the general perforation rate reported for colorectal ESD studies using needle-type knives 2 4 . Although the rate of intraoperative perforation was higher in NJAG compared with JAG (1.9% vs 0.33%), this difference was not statistically significant. In the JAG group, a single case of intraoperative perforation occurred in a lesion with severe fibrosis, requiring surgical intervention. Conversely, three intraoperative perforations were reported in NJAG, all of which were successfully managed endoscopically through clipping, resulting in no further complications. These findings support the safety of using scissor-type knife for colorectal ESD, reinforcing results from previous Japanese studies 11 12 14 . The technique of grasping and cutting tissue with the scissor-type knife minimizes accidental injury to the muscle layer, thereby contributing to safer outcomes. The study results suggest that colorectal ESD with scissor-type knife can be safely performed outside Japan.

In NJAG, a case of delayed perforation was encountered, representing a rare but serious complication previously reported in studies on colorectal ESD with scissor-type knives 19 . Similarly, Yamashina et al. also reported delayed perforation in a case involving the SB Jr. knife, although identifying the underlying cause proved challenging 20 . In our study case, the patient underwent an uneventful ESD procedure in the ascending colon, and the resulting mucosal defect was clipped. However, delayed perforation was confirmed post-procedure by emergency CT. Emergency surgery was performed and the patient recovered well. Upon reviewing the case, we suspected extremely poor bowel preparation might have significantly contributed to the delayed perforation because we confirmed that no obvious perforation was identified during ESD and the post ESD ulcer was totally clipped. We speculated that fecal material could have leaked through gaps between the clips, leading to delayed infection and subsequent damage to the ulcerated bowel wall.

The study results highlight a notably low incidence of delayed bleeding at 0.87%, which stands in contrast to the 3% to 5% range commonly reported in other reported literature 19 21 22 . Interestingly, all instances of delayed bleeding were confined to the JAG group, raising questions about the factors contributing to absence of delayed bleeding in the NJAG. One potential explanation could be routine use of prophylactic clipping at these NJAG institutions, particularly in China. Supporting this, a recent meta-analysis demonstrated that prophylactic clipping reduced post-ESD bleeding events, reporting rates of 2.20% compared with 6.67%, with a notable OR of 0.28 (95% CI 0.13–0.59; *P* < 0.05) 23 . Furthermore, a Japanese study also reported that prophylactic clip closure after colorectal ESD was associated with significantly lower rate of delayed bleeding rate (OR 0.36, 95% CI 0.14–0.90; *P* = 0.029) 24 . These findings further support the safety and acceptability of performing colorectal ESD with a scissor-type knife outside Japan. Our findings indicated that resection speed in the NJAG was significantly slower, at less than half the rate observed in the JAG. This discrepancy can be attributed to several factors. First, a learning curve exists for colorectal ESD using a scissor-type knife, with approximately 37 cases needed to achieve satisfactory resection speed 22 . In addition, non-experts training with colorectal ESD often encounter difficulties in proper entry into the submucosal layer and selection of appropriate dissection strategies, which can lead to prolonged procedure time 25 26 27 . Furthermore, efficient use of a scissor-type knife requires multiple steps, including precise blade rotation and positioning by assistants 13 . In Japan, these assistants are typically residents who are well trained, whereas nurses often fill this role outside Japan, potentially contributing to slower speeds due to varying levels of experience. To mitigate the challenges associated with reduced resection speed when using scissor-type knives, new models and methods are currently in development 13 28 . In addition, traction devices have been shown to accelerate resection speed for colorectal ESD using scissor-type knives and have been particularly beneficial for ESD trainees 26 27 28 29 . Nonetheless, it remains unclear whether these devices will offer the same advantages to non-experts performing ESD with scissor-type knives.

Our result showed that the curative resection rate was not significantly different between NJAG and JAG prior to PSM. However, after matching, the curative resection rate was significantly lower in NJAG (83.3%) compared with JAG (95%), with *P* < 0.01. This observed discrepancy might stem from a pathological bias, because JAG had a higher proportion of T1b cases before matching ( Table 2 ).

The rate of histological complete resection was significantly lower in NJAG compared with JAG, both before (88.9% vs 96.7%; *P* < 0.01) and after matching (88.3% vs 98.3%; *P* < 0.01). This difference may reflect operator experience and inherent learning curve associated with ESD with a scissor-type knife, which is crucial for achieving curative resections 22 . Despite these challenges, it is noteworthy that the histological complete resection rate in institutions outside Japan remains acceptable. Additionally, scissor-type knife has been shown to improve self-completion rates among non-expert operators in Japan 19 . A recent study identified several risk factors associated with incomplete resection, including larger lesion diameter, right-sided colonic location, deeper submucosal invasion, and severe fibrosis 30 . Our study also confirmed that severe submucosal fibrosis and T1b pathology as significant contributors for incomplete resection. Moreover, we identified two additional risk factors associated with incomplete resection in our study: being at institutions outside Japan and slower resection speed. In our opinion these likely underscore the importance of operator experience in achieving histological complete resection 21 .

Hence, although scissor-type knife demonstrated favorable safety in this international scenario, suggesting usage of special device like scissor-type knife helps to achieve the uneventful completion of colorectal ESD, several performance measures, including resection speed and resection outcomes, still need improvement. In the future, ESD training should target the key segments incorporating mucosal flap creation, safe margin assurance, and assistant competency to improve efficiency of colorectal ESD.

This study has several limitations. First, as an observational study, it lacked randomization or procedure standardization, such as consistent settings for the electrosurgical unit. ESD strategies, including use of traction devices, kinds of injection, prophylactic measures such as vessel coagulation and mucosal defect clipping, was left to the discretion of each institution. Second, the comparative study between the two groups, JAG and NJAG, was a subgroup analysis; therefore, the statistical power might not be enough. In addition, there were unbalanced baseline characteristics between the two groups, which we attempted to address through 1:1 PSM. This process resulted in 120 matched pairs, aiming to minimize bias. Third, most operators outside Japan were non-experts, which raises the question whether experts outside Japan would achieve improve efficiency when using scissor-type knives for colorectal ESD. Nevertheless, even with non-expert operators, use of scissor-type knives demonstrated favorable safety and acceptable efficiency.

## Conclusions

In conclusion, this international, multicenter, prospective study provides valuable insight into the safety of colorectal ESD using scissor-type knives, particularly in non-Japanese experts. However, some treatment outcomes, including resection speed and resection rates, were found to be inferior in non-Japanese institutions, emphasizing the need for training and technological advancement. The findings advocate the need for continued exploration and improvement of ESD techniques while using scissor-type knives across diverse clinical healthcare settings while ensuring patient safety and optimal outcome.

## References

[1] Pimentel-Nunes P Libânio D Bastiaansen BAJ Endoscopic submucosal dissection for superficial gastrointestinal lesions: European Society of Gastrointestinal Endoscopy (ESGE) Guideline - Update 2022 Endoscopy 2022 54 591 622 10.1055/a-1811-7025 35523224

[2] Fuccio L Hassan C Ponchon T Clinical outcomes after endoscopic submucosal dissection for colorectal neoplasia: a systematic review and meta-analysis Gastrointest Endosc 2017 86 74 86 10.1016/j.gie.2017.02.024 28254526

[3] Saito Y Yamada M So E Colorectal endoscopic submucosal dissection: Technical advantages compared to endoscopic mucosal resection and minimally invasive surgery Dig Endosc 2014 26 52 61 10.1111/den.12196 24191896

[4] Singh RR Nanavati J Gopakumar H Colorectal endoscopic submucosal dissection in the West: A systematic review and meta-analysis Endosc Int Open 2023 11 E1082 E1091 10.1055/a-2181-5929 38026781 PMC10681808

[5] Draganov PV Wang AY Othman MO AGA Institute Clinical Practice Update: Endoscopic Submucosal Dissection in the United States Clin Gastroenterol Hepatol 2019 17 16 25 e1 30077787 10.1016/j.cgh.2018.07.041

[6] Hayashi N Tanaka S Nishiyama S Predictors of incomplete resection and perforation associated with endoscopic submucosal dissection for colorectal tumors Gastrointest Endosc 2014 79 427 435 24210654 10.1016/j.gie.2013.09.014

[7] Yahagi N Uraoka T Ida Y Endoscopic submucosal dissection using the Flex and the Dual knives Tech Gastrointest Endosc 2011 13 74 78

[8] Toyonaga T Man-I Fujita T The performance of a novel ball tipped Flush knife for endoscopic submucosal dissection: a case control study Aliment Pharmacol Ther 2010 32 908 915 10.1111/j.1365-2036.2010.04425.x 20839389

[9] Homma K Otaki Y Sugawara M Efficacy of novel SB knife Jr examined in a multicenter study on colorectal endoscopic submucosal dissection Dig Endosc 2012 24 117 120 10.1111/j.1443-1661.2012.01266.x 22533765

[10] Akahoshi K Akahane H Motomura Y A new approach: endoscopic submucosal dissection using the Clutch Cutter for early stage digestive tract tumors Digestion 2012 85 80 84 10.1159/000334647 22269283

[11] Kuwai T Yamaguchi T Imagawa H Endoscopic submucosal dissection of early colorectal neoplasms with a monopolar scissor type knife: short- to long-term outcomes Endoscopy 2017 49 913 918 28743145 10.1055/s-0043-113631

[12] Yoshida N Dohi O Inoue K Efficacy of scissor-type knives for endoscopic mucosal dissection of superficial gastrointestinal neoplasms Dig Endosc 2020 32 4 15 10.1111/den.13446 31120558

[13] Kuwai T Tamaru Y Kusunoki R SB Knife Jr: characteristics and tips on how to use Mini-invasive Surg 2022 6 16

[14] Kuwai T Oka S Kamigaichi Y Efficacy and safety comparison of scissor-type knives with needle-type knives for colorectal endoscopic submucosal dissection: a post-hoc propensity score-matched analysis (with videos) Gastrointest Endosc 2022 96 108 117 35247378 10.1016/j.gie.2022.02.042

[15] Gopakumar H Vohra I Reddy Puli S Comparison of scissor-type knife to non-scissor-type knife for endoscopic submucosal dissection: a systematic review and meta-analysis Clin Endosc 2024 57 36 47 38178328 10.5946/ce.2023.122 PMC10834292

[16] Tanaka S Kashida H Saito Y Japan Gastroenterological Endoscopy Society guidelines for colorectal endoscopic submucosal dissection/endoscopic mucosal resection Dig Endosc 2020 32 219 239 10.1111/den.13545 31566804

[17] Hashiguchi Y Muro K Saito Y Japanese Society for Cancer of the Colon and Rectum. Japanese Society for Cancer of the Colon and Rectum (JSCCR) guidelines 2019 for the treatment of colorectal cancer Int J Clin Oncol 2020 25 1 42 10.1007/s10147-019-01485-z 31203527 PMC6946738

[18] Gopakumar H Vohra I Sharma NR Efficacy of scissor-type knife for endoscopic submucosal dissection: a systematic review and meta-analysis Ann Gastroenterol 2023 36 615 623 10.20524/aog.2023.0838 38023980 PMC10662071

[19] Iwatsubo T Takeuchi Y Yamasaki Y Differences in clinical course of intraprocedural and delayed perforation caused by endoscopic submucosal dissection for colorectal neoplasms: A retrospective study Dig Dis 2019 37 53 62 10.1159/000492868 30227392

[20] Yamashina T Takeuchi Y Nagai K Scissor-type knife significantly improves self-completion rate of colorectal endoscopic submucosal dissection: Single-center prospective randomized trial Dig Endosc 2017 29 322 329 27977890 10.1111/den.12784

[21] Yachida T Kobara H Kozuka K Comparison of needle knife versus scissors forceps for colorectal endoscopic submucosal dissection: A prospective randomized study J Clin Med 2023 12 2329 10.3390/jcm12062329 36983328 PMC10056117

[22] Miyakawa A Kuwai T Sakuma Y Learning curve for endoscopic submucosal dissection of early colorectal neoplasms with a monopolar scissor-type knife: use of the cumulative sum method Scand J Gastroenterol 2020 55 1234 1242 32853052 10.1080/00365521.2020.1807597

[23] Jiang W Cen L Dong C Prophylactic clipping to prevent delayed bleeding and perforation after endoscopic submucosal dissection and endoscopic mucosal resection: A systematic review and meta-analysis J Clin Gastroenterol 2022 56 643 653 10.1097/MCG.0000000000001721 35648969

[24] Miyakawa A Kuwai T Sakuma Y The efficacy of prophylactic clip closure of mucosal defects after colorectal endoscopic submucosal dissection on delayed bleeding Scand J Gastroenterol 2021 56 1236 1242 10.1080/00365521.2021.1953129 34362282

[25] Chiba H Ohata K Ashikari K Effectiveness of strategy-focused training in colorectal endoscopic submucosal dissection: A retrospective observational study Dig Dis Sci 2024 69 2370 2380 10.1007/s10620-024-08430-9 38662160

[26] Chang MC Chen WC Yu HC Diving, lifting, and horizontal dissection followed by loop-clip traction (DLH+T) can facilitate mucosal flap creation during colorectal ESD Surg Endosc 2022 36 7811 7817 35648212 10.1007/s00464-022-09324-5

[27] Yang B Yan P Li X Effects of traction methods in inexperienced endoscopists during colorectal endoscopic submucosal dissection Scand J Gastroenterol 2023 58 1056 1063 36941781 10.1080/00365521.2023.2191766

[28] Tamaru Y Kuwai T Miyakawa A Efficacy of a traction device for endoscopic submucosal dissection using a scissor-type knife: A randomized controlled trial Am J Gastroenterol 2022 117 1797 1804 10.14309/ajg.0000000000002019 36191269

[29] Inoue K Yoshida N Dohi O Effects of the combined use of a scissor-type knife and traction clip on endoscopic submucosal dissection of colorectal tumors: a propensity score-matched analysis Endosc Int Open 2021 9 E1617 E1626 10.1055/a-1535-0786 34790523 PMC8589530

[30] Gu F Jiang W Zhu J Risk factors for unsuccessful colorectal endoscopic submucosal dissection: A systematic review and meta-analysis Dig Liver Dis 2024 56 1288 1297 10.1016/j.dld.2023.11.030 38071178

